# Evaluation of OVOL1 and Filaggrin immunohistochemical expression and clinical relevance in psoriasis

**DOI:** 10.1186/s13000-024-01491-4

**Published:** 2024-06-21

**Authors:** Aiat Shaban Hemida, Mostafa Ahmed Hammam, Aya Ahmed Swilam, Wafaa Ahmed Shehata

**Affiliations:** 1 Pathology Department, Faculty of Medicine, Menoufia University, Shebin El Kom, Shebin El Kom, Egypt; 2 Department of Dermatology, Andrology and Venereology, Faculty of Medicine, Menoufia University, Shebin El Kom, Egypt; 3 Department of Dermatology, Andrology and Venereology, Shebin El Kom Teaching Hospital, Menoufia, Egypt

**Keywords:** Psoriasis, OVOL1, Filaggrin, Immunohistochemistry

## Abstract

**Background:**

Psoriasis is a disease of overactive immune system. OVOL1 and Filaggrin have been associated with many inflammatory skin lesions. To the best of our knowledge, the correlation between OVOL1 and Filaggrin in psoriasis was not previously investigated. This work aims to search the immunohistochemical expression and correlation between OVOL1 and Filaggrin in psoriasis.

**Materials and methods:**

Slides cut from paraffin blocks of 30 psoriasis cases and 30 control subjects were stained with OVOL1 and Filaggrin. Clinicopathological data were correlated with the results of staining.

**Results:**

OVOL1 and Filaggrin expression in epidermis showed a significant gradual reduction from normal skin to peri-lesional and psoriasis biopsies (*P* < 0.001). In contrast, psoriasis dermis showed a significant overexpression of OVOL1 in inflammatory cells in relation to peri-lesional biopsies (*P* < 0.002). OVOL1 demonstrated a significant direct correlation with Filaggrin expression in psoriasis (*r* = 0.568, *P* < 0.004). OVOL1 and Filaggrin expression in psoriasis skin epidermis demonstrated a statistically significant negative correlation with PASI score.

**Conclusion:**

OVOL1 and Filaggrin might be involved in psoriasis-associated inflammation and skin hyperproliferation. OVOL1 might have a protective barrier function in the skin and could be used to stratify progressive disease. Filaggrin may play a role in progression of psoriasis. OVOL1 inhibition could be considered in suppression of Filaggrin function. OVOL1 agonists may be beneficial in psoriasis treatment.

## Introduction

Psoriasis is disease of dysregulated immune system that affects 0.33- 0.6% in various races [1]. It is considered a global problem that affects around 125 million people worldwide [2]. Psoriasis affected 3.0% of adults as per Bayesian analysis of expert estimates [3].

Psoriasis is a disease of systemic inflammation with overactive immune system and was associated with increasing incidence of comorbid conditions. Psoriasis histopathology showed proliferation and disturbed differentiation of epidermis, tortuous and dilated vessels and an inflammatory infiltrate of dermis. The cause of psoriasis was unknown, but it can be dysregulated inflammation, environmental and genetic associations [4]. Psoriasis was partially controlled with treatment and currently no cure is established [5]. Psoriasis remained a lifelong burden that impaired socioeconomic stability and quality of life.

OVOL1, vertebrate homologs of *Drosophila* OVO, was found in various epithelial cells, including epidermis, renal epithelium and testes [6]. OVOL1 function in maintaining differentiated epidermal cells and hair follicles. It was normally expressed in the suprabasal intermediate and spinous cells of skin epidermis that bear the power of proliferation. In addition, OVOL1 restricted proliferation of progenitor cells in epidermis and regulated the needed equilibrium between proliferation and differentiation of these cells. In its absence, it was found that keratinocytes failed to respond to extrinsic signals that inhibit their growth in culture [7].

To the best of our knowledge, only two studies searched if OVOL1 shared in psoriasis development [8, 9]. Sun et al., (2020) study demonstrated that OVOL1 regulated a protective function and prevented psoriasis-like inflammation [8]. In addition, Dragan et al., (2022), the same team of the previous study, concluded that in mice, OVOL1 was altered by germline Ovol1 deletion and this inhibited the epidermal barrier, and potentiated psoriasis-like skin inflammation by promoting neutrophil attraction with formation of multiple abscesses [9]. Therefore, the expression and role of OVOL1 in psoriasis remained obscured and required further assessment.

Filaggrin, is a highly abundant keratin filament associated protein that is present in the outermost layers of the epidermis. It had been isolated from the stratum corneum and found to be important for development of the cornified cell envelope. Filaggrin is important to maintain the epidermal barrier and hydration. In addition, Filaggrin is a marker of terminal differentiation of the epidermis. Mutations that inhibited the FLG gene predisposed to both ichthyosis vulgaris and atopic dermatitis. In human and mice, decreased Filaggrin expression was associated with abnormalities in epidermal barrier and involved in psoriasis pathogenesis [10]. On the contrary, previous data suggested that mutations in Filaggrin were not involved in genetic predisposition to psoriasis [11]. Thus, it remains to be seen if Filaggrin is involved in psoriasis pathogenesis.

The relation between OVOL1 and Filaggrin is unclear. To the best of our knowledge, no previous studies evaluated the correlation between OVOL1 and Filaggrin in psoriasis. This research aimed to demonstrate the expression of OVOL1 and Filaggrin in plaque psoriasis and investigate the possible relation between them.

## Materials and methods

### Study cohort

Thirty patients diagnosed with plaque psoriasis irrespective of age and sex and 30 control subjects were included from Dermatology Outpatient Clinic during the period between September 2021 and March 2023. Thirty age and sex matched healthy subjects were selected as a control group.

Participants in this work approved a written consent according to the Local Ethical Committee of university policies that matched Helsinki Declaration of 1975 (revised in 2000) (IRB approval number: 5/2021 DERMA19). Before taking the biopsy, selected participants stopped topical medications for two weeks and systemic medications for one month.

Onset of psoriasis was categorized as early onset (< 40 years old) and late onset (> 40 years old). Participants were examined to assess psoriasis and its severity using Psoriasis Area and Severity Index (PASI score) and they were categorized into mild (< 10), moderate (10–20) and severe (> 20) cases [12].

### Skin biopsies

Skin biopsies were taken with 3.5-millimeter punch from the psoriatic lesion and peri-lesional skin (within 5 cm of psoriatic lesion) of each patient. Control skin biopsies were taken at plastic surgery department from healthy skin removed in operations.

Tissue processing was done and paraffin blocks were prepared; one slide stained by hematoxylin and eosin was examined to assess histopathological parameters, and two slides for immunostaining were prepared.

### Immunohistochemistry

Streptavidin-biotin‐amplified system was followed. Anti- OVOL1 antibody (0.1 mL concentrated and diluted 1:100, Rabbit polyclonal antibody) (Abbkine, Inc., China. Co., catalog# ABP55452) and anti- Filaggrin antibody (Abcam Inc., Cambridge, UK. FLG-1562,, mouse monoclonal antibody, 1:200) (catalog# ab218397) were the primary antibodies. Heat retrieval was done with citrate buffer for the two primary antibodies. Positive control slides of placental tissue and normal skin tissue for OVOL1 and Filaggrin respectively, in addition to negative control slides were checked in each run.

### Interpretation of OVOL1 immunohistochemical staining

The expression of OVOL1 and Filaggrin was considered positive when ≥ 1% staining [13]. OVOL1 showed brown nuclear or nucleocytoplasmic staining of keratinocytes [14]. The expression of Filaggrin showed brown nuclear or nucleocytoplasmic staining of stratum corneum, stratum granulosum and extending to whole epidermal layers [15]. OVOL1 and Filaggrin were evaluated in psoriatic lesions, perilesional biopsies, and normal control groups. Percent of positive cells was registered at 100× field. Weak, moderate or strong staining intensity were recorded. H-score was estimated, (depending on percent and intensity of expression), using the equation: H-score = 1 × (percent of weakly stained cells) + 2 × (percent of moderately stained cells) + 3 × (percent of strongly stained cells). The final score value range from 1 to 300 [16].

### Statistical analysis

IBM statistical package for the social sciences (SPSS) software—version 20 for Windows (*SPSS Inc., Chicago, Illinois, USA*) was used. Descriptive statistics were demonstrated as mean, standard deviation (SD), range, numbers and percentages. Analytical statistics were used to evaluate the association between clinicopathological parameters and psoriasis. Statistical significance documented as a probability level of *p* ≤ 0.05.

## Results

### Histopathological data of psoriasis patients

Histopathological data of psoriasis cases were presented in (Table 1).

**Table 1:** Histopathological findings of psoriasis patients

Studied variables	*N*	%
Epidermis		
**Acanthosis** Mild Moderate Marked	4 10 16	13.4 33.3 53.3
**Hyperkeratosis** Mild Moderate Marked	10 5 15	33.3 16.7 50.0
**Parakeratosis** Mild Moderate Marked	16 12 2	53.3 40.0 6.7
**Suprapapillary thining** Yes No	29 1	96.7 3.3
**Munro’s microabscesses** Present Absent	2 28	6.7 93.3
**Spongiform pustules of Kogoj** Present Absent	2 28	6.7 93.3
**Dermis**		
**Dilated blood vessels in papillary dermis** Present Absent	30 0	100.0 0.0
**Perivascular inflammation** Mild Moderate Severe	9 8 13	30.0 26.7 43.3

N: Number %: Percent

### Comparison between psoriasis lesions, peri-lesional, and normal groups as regards OVOL1 and Filaggrin immunostaining

**Fig. 1:**
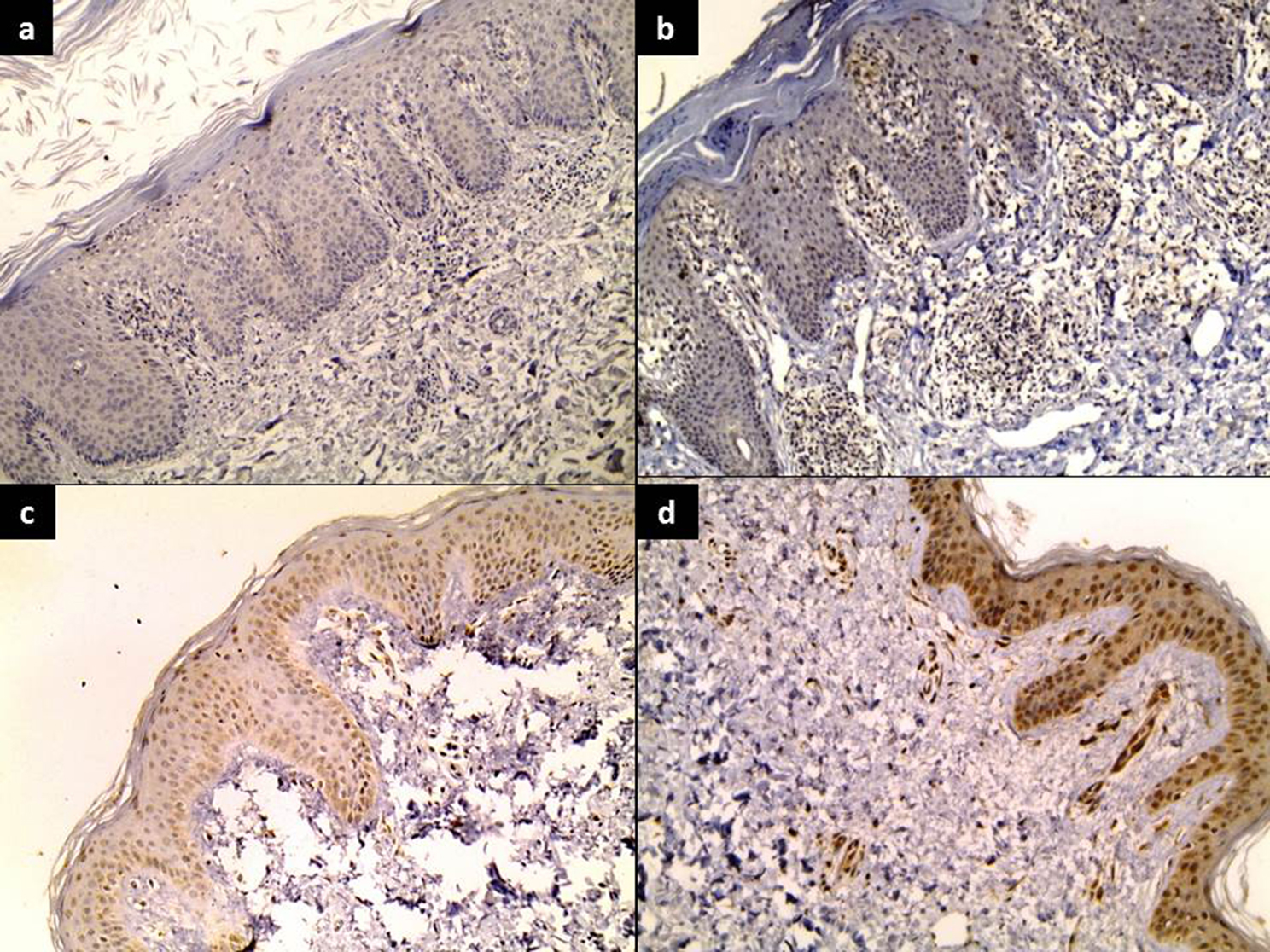
OVOL1 showed (**a**) A negative expression in lesional skin (IHC ×100) (**b**) mild expression in epidermis, strong expression in dermal inflammatory cells of lesional skin (IHC staining ×100), (**c**) moderate expression in peri-lesional skin (IHC staining ×200), (**d**) strong expression in control skin (IHC staining ×100)

OVOL1 immunostaining showed a significant gradual reduction in its expression in epidermal keratinocytes from controls (174.63 ± 34.52) to peri-lesional (83.33 ± 54.14) and lesional (65.71 ± 45.02) skin (*P* < 0.001). In contrast, there was a significant overexpression of OVOL1 in inflammatory cells in psoriasis dermis (169.61 ± 68.85) when compared to peri-lesional biopsies (112.14 ± 34.01) (*P* < 0.002) (Table 2) (Fig. 1).

**Table 2:** Comparison between controls, psoriatic skin (lesional and peri-lesional) regarding OVOL1 expression

	Lesional (*n* = 30)		Peri-lesional (*n* = 30)		Control (*n* = 30)		Test of Sig. (*p*)	Post Hoc test
	*N*	%	*N*	%	*N*	%		
OVOL1 expression in Epidermis								
Negative	16	53.3	3	10.0	0	0.0	χ^2^= 28.955^*^ (*P* < 0.001^*^)	p_1_ < 0.001^*^ p_2_ < 0.001^*^ p_3_ = _0_.237
Positive	14	46.7	27	90.0	30	100.0		
**OVOL1 Intensity in Epidermis**								
Mild	8	57.1	14	51.9	1	3.3	χ^2^= 23.565^*^ (*P* < 0.001^*^)	^MC^p_1_=0.902, ^MC^p_2_<0.001^*^, p_3_ < 0.001^*^
Moderate	4	28.6	10	37.0	17	56.7		
Strong	2	14.3	3	11.1	12	40.0		
**OVOL1% of positive cells in Epidermis**								
X ± SD.	41.43 ± 17.91		48.52 ± 15.86		76.67 ± 11.84		H 37.471* (< 0.001*)	p_1_ = 0.375, p_2_ < 0.001^*^, p_3_ < 0.001^*^
Median	35.0		50.0		80.0			
**OVOL1 H- score in Epidermis**								
X ± SD.	65.71 ± 45.02		83.33 ± 54.14		174.63 ± 34.52		H 37.466^*^ (< 0.001^*^)	p_1_ = 0.511, p_2_ < 0.001^*^, p_3_ < 0.001^*^
Median	50.0		70.0		180.0			
**OVOL1 expression in Dermis**								
Negative	4	13.3	16	53.3	15	50.0	χ^2^= 12.436^*^ (P 0.002^*^)	p_1_ = 0.001^*^ p_2_ = 0.002^*^ ^FE^p_3_=0.796
Positive	26	86.7	14	46.7	15	50.0		
**OVOL1 Intensity in Dermis**								
Mild	4	15.4	1	7.1	0	0.0	χ^2^= 7.763 (^MC^*p*= 0.074)	^MC^p_1_=0.041^*^ ^MC^p_2_=0.358 ^MC^p_3_ = 0.132
Moderate	8	30.8	10	71.4	7	46.7		
Strong	14	53.8	3	21.4	8	53.3		
**OVOL1% of positive cells in Dermis**								
X ± SD.	69.23 ± 12.30		53.57 ± 13.93		68.0 ± 10.14		H 10.972^*^ (0.004^*^)	p_1_ = 0.001^*^ p_2_ = 0.753 p_3_ = 0.010^*^
Median	70.0(60.0–80.0)		50.0(50.0–70.0)		70.0(60.0–70.0)			
**OVOL1 H- score in Dermis**								
X ± SD.	169.61 ± 68.85		112.14 ± 34.01		170.0 ± 31.85		H 11.612^*^ (0.003^*^)	p_1_ = 0.002^*^ p_2_ = 0.847 p_3_ = 0.003^*^
Median	180.0(120.0–210.0)		100.0(90.0–140.0)		180.0(145.0–195.0)			

N: Number X: Mean %:Percent SD: **Standard deviation**

χ^2^:**Chi square test** MC: **Monte Carlo** E: **Fisher Exact**

H: H for **Kruskal Wallis test**

Pairwise comparison bet. each 2 groups was done using **Post Hoc Test (Dunn’s for multiple comparisons test)**

p: p value for comparing between the three studied groups

p_1_: p value for comparing between **Lesional** and **Peri-lesional**

p_2_: p value for comparing between **Lesional** and **Control**

p_3_: p value for comparing between **Peri-lesional** and **Control**

*: Statistically significant at *p* ≤ 0.05

**Fig. 2:**
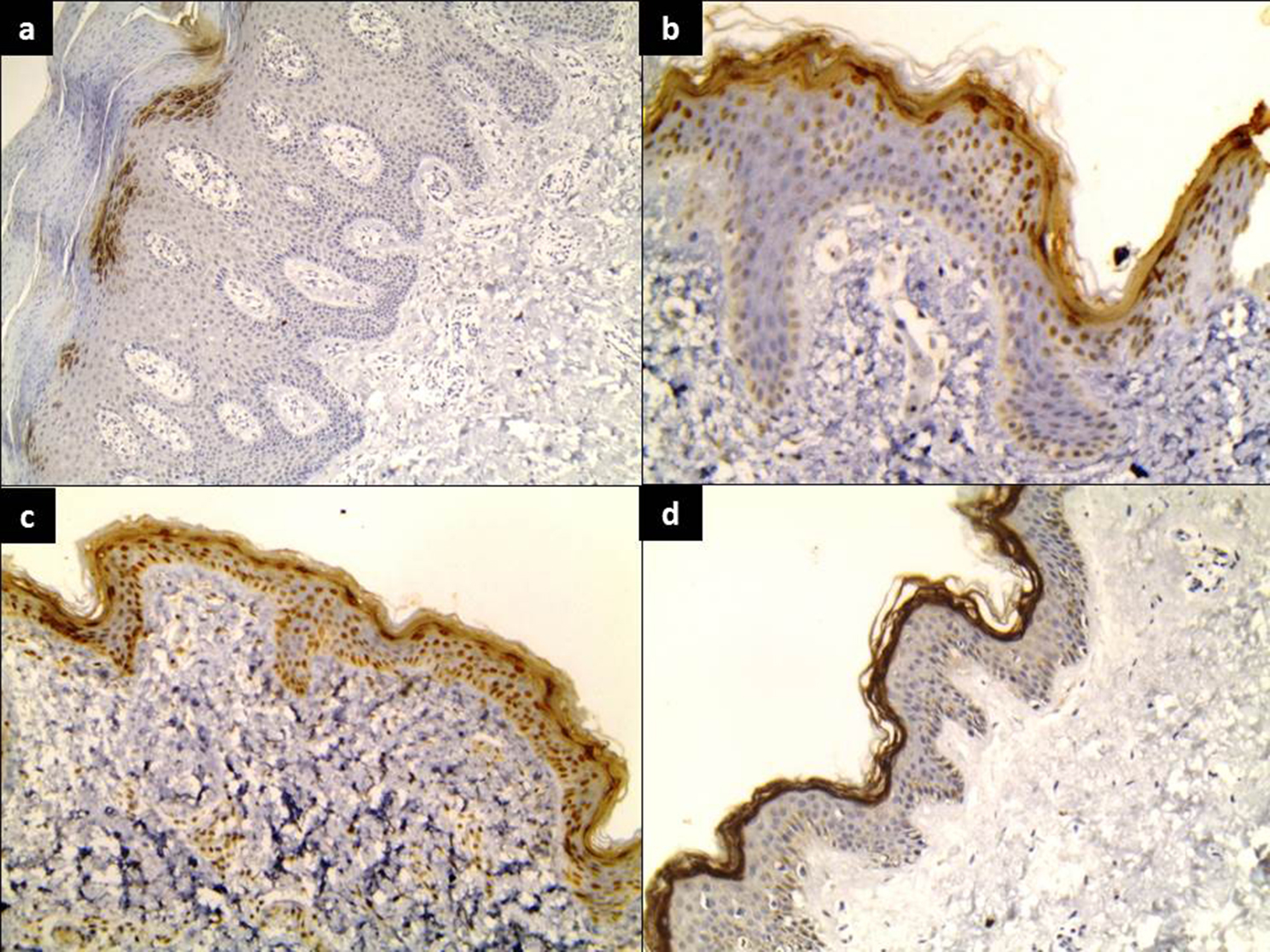
Filaggrin showed (**a**) A negative expression in lesional skin (IHC ×100) (**b**) mild expression in peri-lesional skin (IHC staining ×200), (**c**) strong expression in control skin(corneal, granular and spinous layers) (IHC staining ×100), (**d**) strong expression in control skin (corneal and granular layers) (IHC staining ×100)

Filaggrin immunostaining showed a significant gradual reduction in its expression mainly in epidermal corneal and granular layers from controls (177.63 ± 39.10) to peri-lesional (112.69 ± 52.04) and lesional (104.38 ± 48.02) skin (*P* < 0.001). In addition, there was a significant gradual reduction of Filaggrin in dermal blood vessels and inflammatory cells from controls (191.11 ± 34.16) to peri-lesional (75.45 ± 26.22) and lesional (42.0 ± 13.04) skin (*P* < 0.001) (Table 3) (Fig. 2).

**Table 3:** Comparison between controls, psoriatic skin (lesional and peri-lesional) regarding Filaggrin expression

	Lesional (*n* = 30)			Peri-lesional (*n* = 30)			Control (*n* = 30)		Test of Sig. (*p*)	Post Hoc test
	*N*	%		*N*		%	*N*	%		
Filaggrin expression in Epidermis										
Negative	14	46.7		4		13.3	0	0.0	χ^2^= 21.667^*^ (*P* < 0.001^*^)	p_1_ = 0.005^*^ p_2_ < 0.001^*^ ^FE^p_3_=0.112
Positive	16	53.3		26		86.7	30	100.0		
**Filaggrin Intensity in Epidermis**										
Mild	3	18.8		8		30.8	0	0.0	χ^2^= 15.150^*^ (*P* < 0.001^*^)	p_1_ = 0.198, p_2_ < 0.001^*^ ^FE^p_3_=0.112
Moderate	5	31.3		12		46.2	12	40.0		
Strong	8	50.0		6		23.1	18	60.0		
**Filaggrin percent of positive cells in Epidermis**										
X ± SD.	46.25 ± 15.44			57.69 ± 13.06			71.0 ± 14.23		H 22.450^*^ (< 0.001^*^)	p_1_ = 0.047^*^, ^MC^p_2_=0.071, ^MC^p_3_<0.001^*^
Median	50.0			60.0			70.0			
**Filaggrin H- score in Epidermis**										
	(*n* = 16)			(*n* = 26)			(*n* = 30)			
X ± SD.	104.38 ± 48.02			112.69 ± 52.04			177.63 ± 39.10		H 24.583^*^ (< 0.001^*^)	p_1_ = 0.739, p_2_ < 0.001^*^, p_3_ < 0.001^*^
Median	85.0			120.0			180.0			
Median	50.0			70.0			180.0			
**Filaggrin expression in Dermis**										
Negative	25	83.3	19		63.3		11	36.7	χ^2^= 13.839^*^ (P 0.001^*^)	p_1_ = 0.080 p_2_ < 0.001^*^ p_3_ = 0.039^*^
Positive	5	16.7	11		36.7		19	63.3		
**Filaggrin Intensity in Dermis**										
Mild	4	80.0	7		63.6		0	0.0	χ^2^= 27.646^*^ (^MC^*p*<0.001^*^)	^FE^p_1_=1.000 p_2_ < 0.001^*^ ^FE^p_3_<0.001^*^
Moderate	1	20.0	4		36.4		5	26.3		
Strong	0	0.0	0		0.0		14	73.7		
**Filaggrin percent of positive cells in Dermis**										
X ± SD.	36.0 ± 8.94		57.27 ± 12.72				72.11 ± 13.98		H 16.560^*^ (< 0.001^*^)	p_1_ = 0.058 p_2_ < 0.001^*^ p_3_ = 0.017^*^
Median	30.0(30.0–40.0)		60.0(50.0–60.0)				70.0(60.0–80.0)			
**Filaggrin H- score in Dermis**										
X ± SD.	42.0 ± 13.04		75.45 ± 26.22				191.11 ± 34.16		H 26.899^*^ (< 0.001^*^)	p_1_ = 0.226 p_2_ < 0.001^*^ p_3_ < 0.001^*^
Median	40.0(30.0–50.0)		60.0(60.0–90.0)				180.0(60.0–70.0)			

N: Number X: Mean %:Percent SD: **Standard deviation**

χ^2^:**Chi square test** MC: **Monte Carlo** E: **Fisher Exact**

H: H for **Kruskal Wallis test**

Pairwise comparison bet. each 2 groups was done using **Post Hoc Test (Dunn’s for multiple comparisons test)**

p: p value for comparing between the three studied groups

p_1_: p value for comparing between **Lesional** and **Peri-lesional**

p_2_: p value for comparing between **Lesional** and **Control**

p_3_: p value for comparing between **Peri-lesional** and **Control**

*: Statistically significant at *p* ≤ 0.05

### Correlation between OVOL1 and Filaggrin in the investigated cases

**Fig. 3:**
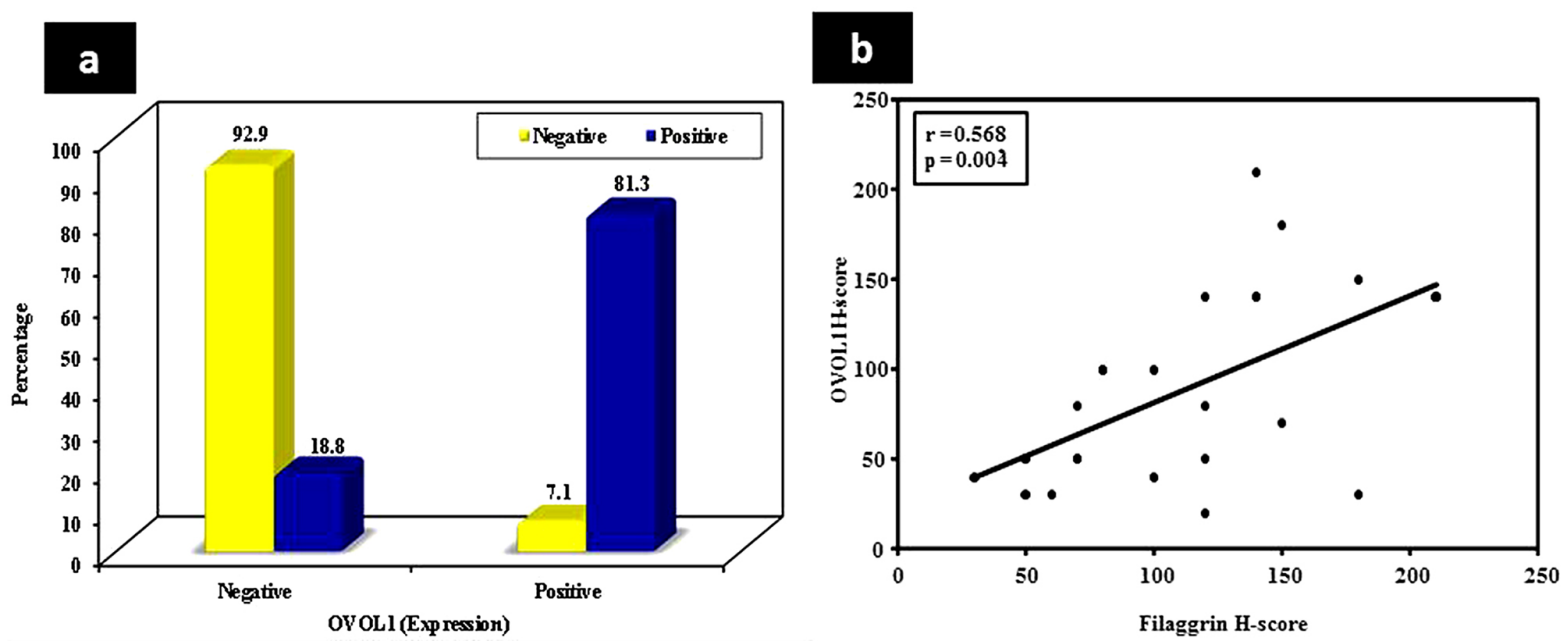
(**a**) A significant direct relationship between OVOL1 and Filaggrin regarding epidermal expression in lesional skin (**b**) A significant direct correlation between OVOL1 and Filaggrin regarding epidermal H-score in peri-lesional skin of the studied cases

OVOL1 epidermal expression demonstrated a significant direct relationship with Filaggrin expression in psoriatic skin (*P* < 0.001) (Fig. 3a).

There was a statistically significant direct correlation between OVOL1 and Filaggrin regarding epidermal H-score in peri-lesional skin of the studied cases (*r* = 0.568, *P* < 0.004) (Fig. 3b).

### Relationship between OVOL1 expression in psoriasis epidermis and clinicopathological findings of psoriasis group

**Fig. 4:**
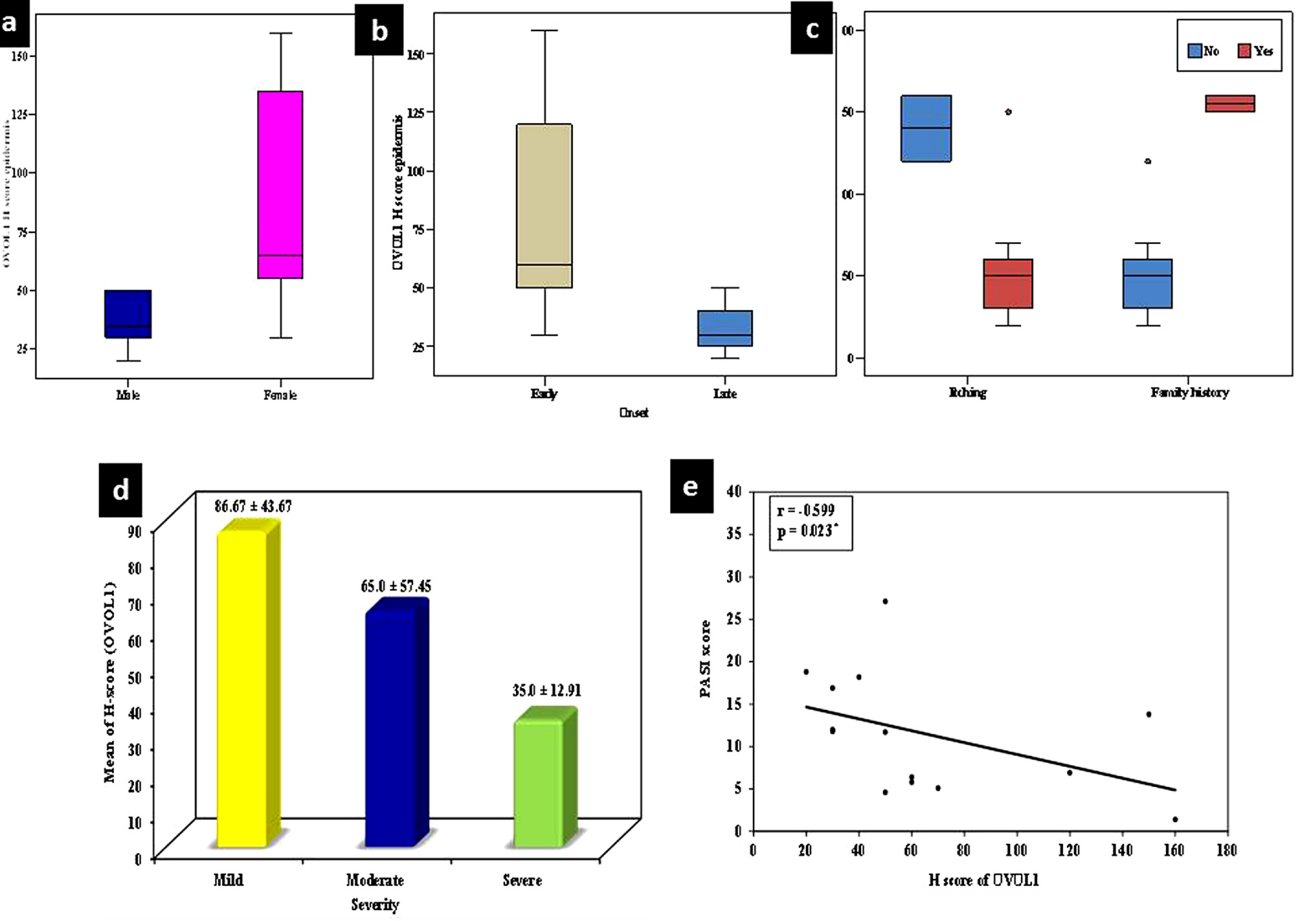
High OVOL1 H-score in lesional epidermis was associated with (**a**) female gender, (**b**) early onset of the disease, absence of itching & (**c**) positive family history of similar condition in the studied cases, (**d**) severity of the disease. (**e**) Significant *negative* correlation between epidermal H score of OVOL1 in lesional skin and PASI score

High OVOL1 H-score in lesional epidermis was associated with female gender (*p* < 0.013), early onset of the disease (*p* < 0.024), absence of itching (*p* < 0.044) & positive family history of similar condition (*p* < 0.022) in the studied cases (Fig. 4a-c).

Epidermal mean H-score of OVOL1 in lesional skin showed a significant relationship with severity of the disease being higher in mild disease (*p* < 0.044) (Fig. 4d). Epidermal mean H-score of OVOL1 in lesional skin showed a significant *negative* correlations with PASI score categories mild (< 10), moderate (10–20) and severe (> 20) cases) **r**_**s**_ = -0.599, *p* < 0.023) (Fig. 4e).

None of the investigated histopathological findings showed a significant relationship with epidermal OVOL1 expression in lesional skin.

### Relationship between OVOL1 expression in psoriasis dermal fibroblasts and inflammatory cells and clinicopathological findings of psoriasis group

**Fig. 5:**
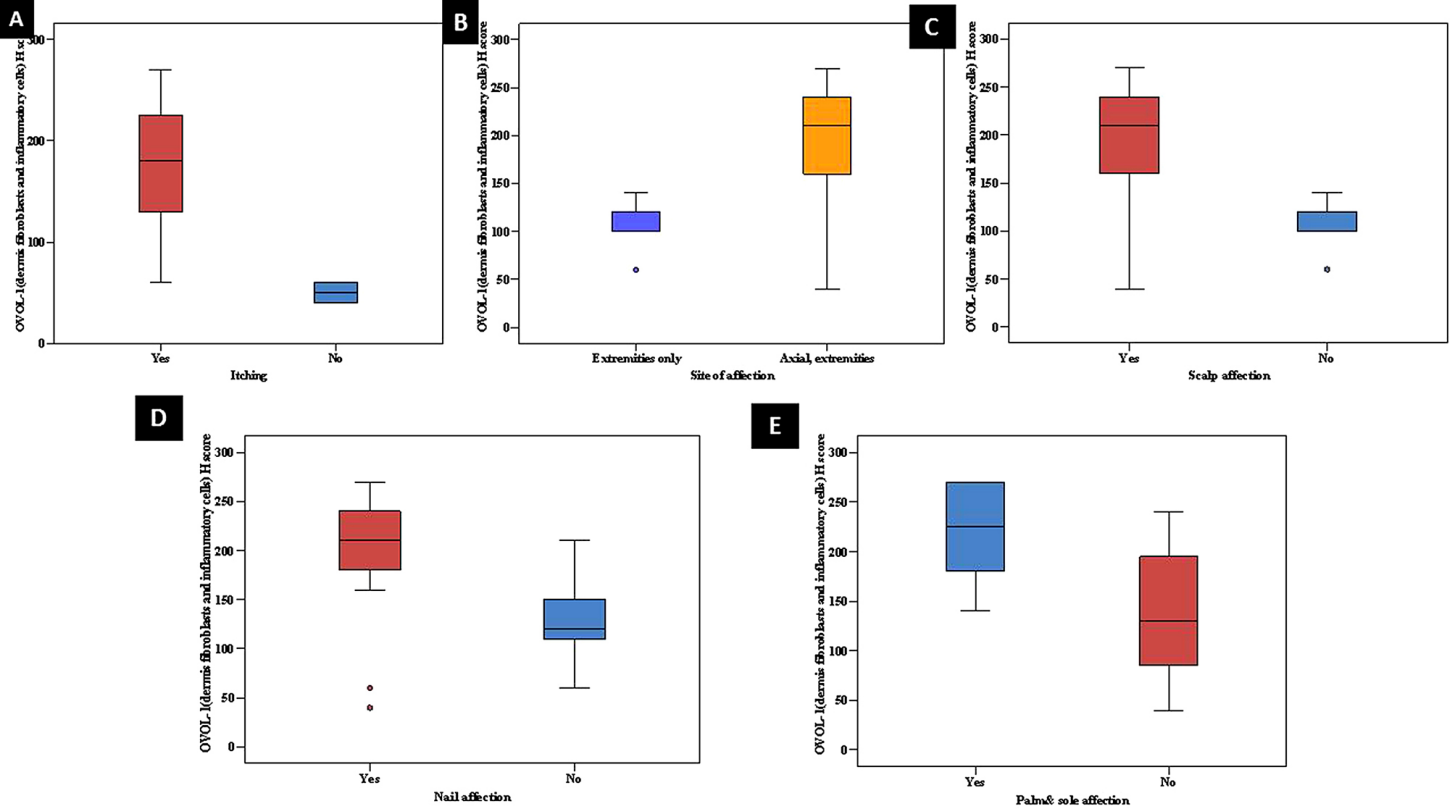
Statistically significant relationships between high dermal mean H-score of OVOL1 in lesional skin and (**a**) presence of itching, (**b**) axial and extremities affection of the disease, (**c**) scalp affection, (**d**) nail affection, and (**e**) palm and sole affection

Statistically significant relationships between high dermal mean H-score of OVOL1 in psoriasis skin and presence of itching (*p* < 0.006), axial and extremities affection of the disease (*p* < 0.017), scalp affection (*p* < 0.015), nail affection (*p* < 0.004) and palm and sole affection (*p* < 0.003) were demonstrated (Fig. 5).

**Fig. 6:**
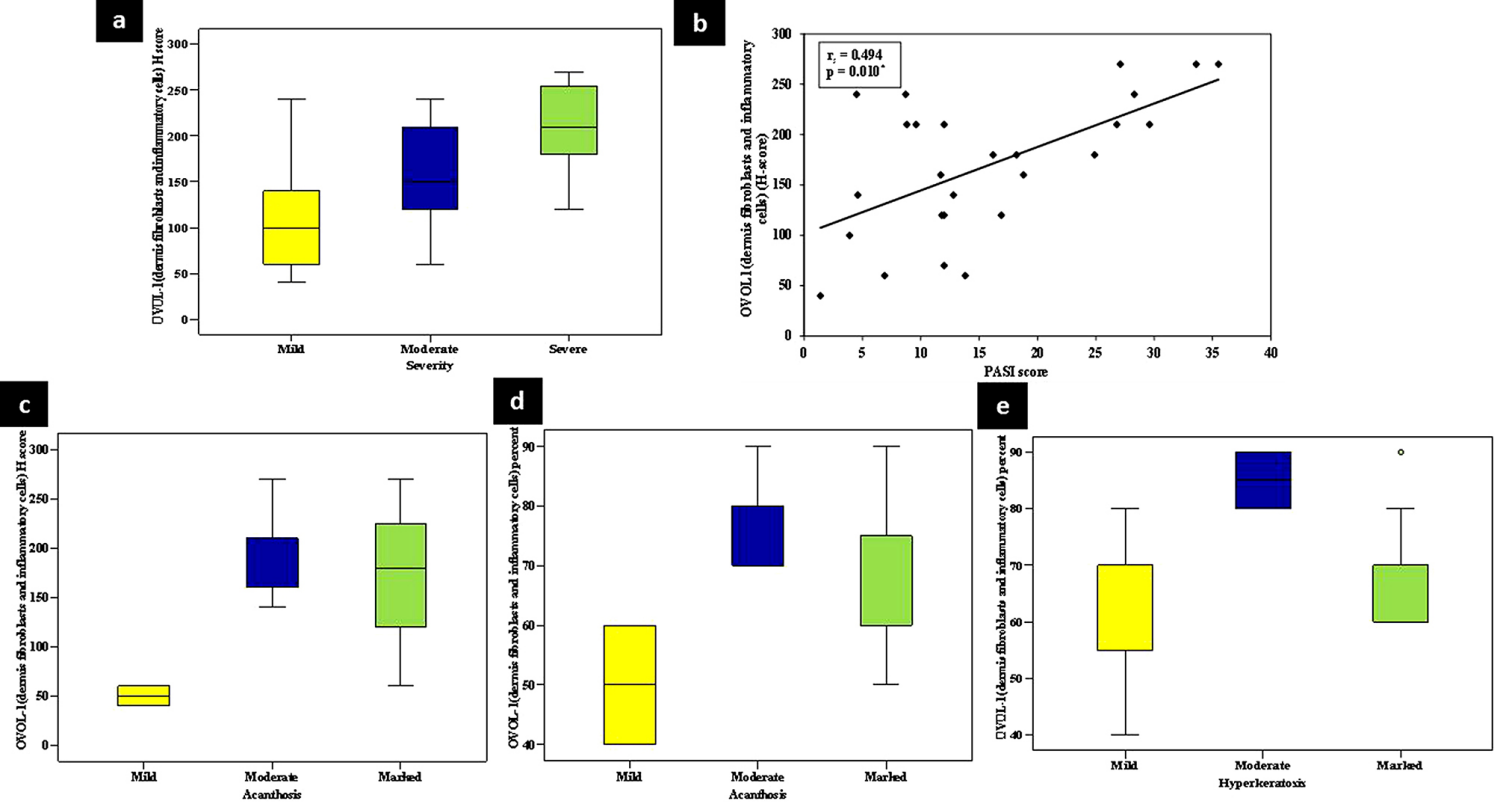
(**a**) A significant relationship between high dermal OVOL1 H-score in lesional skin and marked disease severity. (**b**) Direct positive correlation between dermal OVOL1H-score in lesional skin and PASI score. A significant relationship between high dermal OVOL1 H-score in lesional skin and (**c**) marked acanthosis. A significant relationship between dermal OVOL1 percent of expression and (**d**) marked acanthosis, (**e**) marked hyperkeratosis

There was a statistically significant relationship between high dermal mean H-score of OVOL1 in psoriasis skin and marked severity of disease in the studied cases (*p* < 0.042). In addition, PASI score categories mild (< 10), moderate (10–20) and severe (> 20) cases showed a significant direct positive correlation with dermal H-score of OVOL1 in psoriasis skin (**r**_**s**_ =0.494, *p* < 0.010) (Fig. 6).

Regarding histopathological data, a statistically significant relationship was found between high dermal mean H-score of OVOL1 in psoriasis skin and increased acanthosis in the studied cases (*p* < 0.038). In addition, high dermal mean percent of positive cells of OVOL1 in psoriasis skin showed a significant relationship with increased acanthosis (*p* = 0.030) and hyperkeratosis (*p* = 0.018) (Fig. 6).

### Relationship between Filaggrin expression in psoriasis epidermis and clinicopathological findings of psoriasis group

**Fig. 7:**
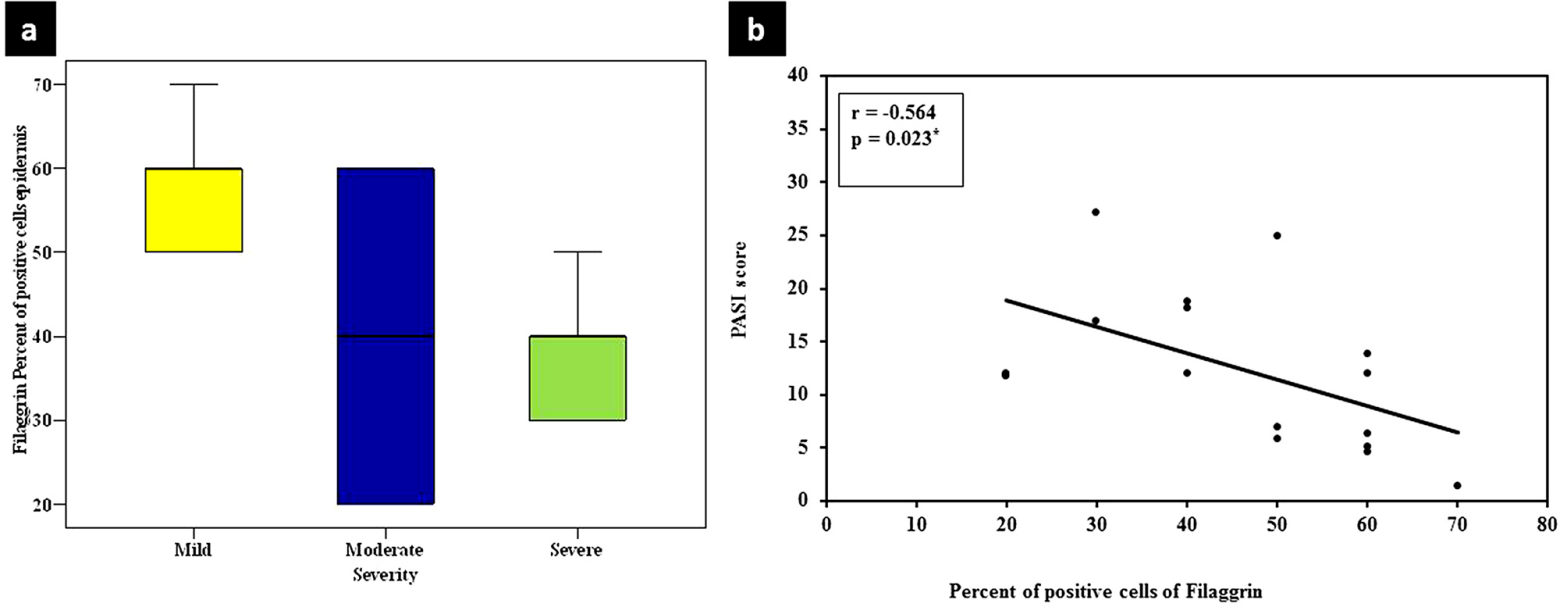
(**a**) A significant relationship between high epidermal mean percent of positive cells of Filaggrin in lesional skin and mild psoriasis severity. (**b**) Negative correlation between percent of positive cells of Filaggrin in epidermis of lesional skin and PASI score

There was a statistically significant relationship between high epidermal mean percent of positive cells of Filaggrin in lesional skin and mild psoriasis severity (*p* = 0.045). In addition, a significant negative correlation was found between percent of positive cells of Filaggrin in epidermis of lesional skin and PASI score categories mild (< 10), moderate (10–20) and severe (> 20) of the studied cases (**r**_**s**_ =-0.564, *p* = 0.023) (Fig. 7).

Regarding histopathological data, there was a statistically non-significant relationship between epidermal Filaggrin expression in lesional skin and histopathological findings of the studied cases.

## Discussion

To the best of our knowledge, the correlation between OVOL1 and Filaggrin in psoriasis has not been previously investigated. In atopic dermatitis, Tsuji et al., (2017) found that OVOL1 levels were correlated with Filaggrin mRNA and protein expression and that aryl hydrocarbon receptor- induced Filaggrin overexpression is blocked when OVOL1-inhibited [17]. In plant, it was demonstrated that Rhodiola crenulata root extract induced Filaggrin overexpression in an aryl hydrocarbon receptor-OVOL1-dependent fashion [18].

In the present work, we recorded a significant stepwise reduction in keratinocytes OVOL1 expression from controls to peri-lesional and psoriasis skin. OVOL1 restricted proliferation of progenitor cells in epidermis and regulated the needed equilibrium between proliferation and differentiation of these cells [7]. Thus, reduction of OVOL1 in psoriatic skin epidermis in this study might be the cause of epidermal hyperproliferation; which is the main feature of psoriasis.

Regarding OVOL1 expression in inflammatory cells of lesional dermis, there was a significant stepwise overexpression when compared to peri-lesional skin. Sun and his team found similar results. They investigated the in vivo function of Ovol1/OVOL1 in psoriasis inflammation and declared a protective role for OVOL1 in preventing psoriasis-like inflammation. OVOL1 loss leads to flaring of psoriasis-like inflammation and proliferation of epidermis in response to imiquimod (IMQ) [8]. In addition, this team continued their research and investigated the barrier function of OVOL1 in psoriasis in skin of mice. They concluded that in mice, OVOL1 was altered by germline Ovol1 deletion and this inhibited the epidermal barrier, and potentiates psoriasis-like skin inflammation by promoting neutrophil attraction with formation of multiple abscesses [9]. These results can suggest a role of OVOL1 in psoriasis- associated inflammation and epidermal proliferation.

In this study, a significant stepwise reduction in Filaggrin immunostaining from normal to peri-lesional and psoriasis skin was recorded. In addition, there was a significant gradual reduction of Filaggrin in dermal blood vessels and inflammatory cells from normal to peri-lesional and psoriasis skin. Previous studies have declared decreased expression of Filaggrin in psoriatic lesional skin than normal skin [10, 19, 20]. However, Zhao et al., (2007) suggested that Filaggrin are unlikely to be involved in psoriasis pathogenesis [11].

Notably, it was documented that Filaggrin is a key player in terminal differentiation of keratinocytes, formation of epidermal barrier, hydration and modulating inflammatory responses [21]. In addition, increased expression of Filaggrin was detected after treatment of psoriasis, followed by the down-regulation of proinflammatory factors, improvement of the skin barrier and remission [22]. Taken together, decreased Filaggrin in psoriatic lesion may suggest its involvement in psoriasis pathogenesis.

Regarding relationship between OVOL1 and Filaggrin epidermal expression in psoriatic skin, our study recorded a significant direct relationship. In addition, there was statistically significant direct correlation between OVOL1 and Filaggrin in peri-lesional skin epidermis. The relation between Filaggrin and OVOL1 in psoriasis is still unclear.

The aryl hydrocarbon receptor (AHR) is a transcription factor expressed in keratinocyte and was suggested to have a significant relationship to psoriasis [23]. In an imiquimod-induced psoriasis model, AHR deficiency increases inflammation of skin [24]. A selective AHR agonist was investigated as an agent that may improve both psoriasis and atopic dermatitis [25]. In atopic dermatitis, it was found that AHR signaling increased the expression of OVOL1, which after its passage to the nucleus increased Filaggrin. In addition, OVOL1 inhibition was involved in Filaggrin reduction, which might be involved in atopic dermatitis pathogenesis [17]. These results could explain the significant direct relationship between OVOL1 and Filaggrin expression in psoriatic skin in our study and that reduced Filaggrin expression in psoriasis might be regulated by the AHR–OVOL1 axis. Therefore, OVOL1 agonist could play a role as a target therapy in psoriasis treatment.

To the best of our knowledge, no previous studies correlated OVOL1 expression in psoriasis with the clinicopathological features. In the current study, high OVOL1 in lesional epidermis was associated with good prognostic parameters as early onset of the disease, absence of itching, positive family history of similar condition and severity of the disease being higher in mild disease. In addition, there was a statistically significant negative correlation between epidermal OVOL1 in psoriasis skin and PASI score. These results confirm the protective barrier function of OVOL1 in the skin epidermis and that decreased its expression in psoriatic epidermis could be used as a sign of progressive disease.

On the contrary, high dermal OVOL1 in lesional skin showed a significant association with presence of itching, axial and extremities affection of the disease, scalp affection, nail affection and palm and sole affection, marked severity of the disease. In addition, there was a direct correlation between dermal OVOL1 in psoriasis skin and PASI score. Regarding histopathological data, high mean percent of positive cells of OVOL1 in psoriasis dermis showed a significant association with increased acanthosis and hyperkeratosis. Confirming these results; Sun et al., (2021) found that the response to IMQ was tailored by loss of *Ovol1* not only in skin epidermis but also in fibroblasts and inflammatory cells. They also concluded that OVOL1 protected the skin barrier by diminishing psoriasis-like inflammation and the associated pathologic changes [8]. These results collectively indicated that reduced epidermal and increased dermal OVOL1 expression were associated with advanced, progressive psoriatic disease.

Regarding association of Filaggrin with the studied clinicopathological parameters, a statistically significant relationship between high Filaggrin in psoriasis skin epidermis and mild psoriasis severity was found. In addition, negative correlation was found between percent of positive cells of Filaggrin in epidermis of lesional skin and PASI score. Therefore, Filaggrin could have a role in progression of psoriasis.

Furue et al. (2019) in genome-wide association meta-analysis studies found that Filaggrin, OVOL1 and IL13 were the three genes most significantly associated with atopic dermatitis among 31 susceptible gene loci reported. They suggested their therapeutic importance [26]. In addition, previous study on atopic dermatitis demonstrated that OVOL1 regulates Filaggrin expression and *Ovol1*-deficient keratinocytes showed reduced expression of Filaggrin in the suprabasal compartment of epidermis [17]. Moreover, Dębińska, 2021 study on restoring Filaggrin deficiency to improve skin barrier function included many therapeutic strategies that could be promising for atopic dermatitis treatment. The gene-based and direct replacement Filaggrin therapy was not available, thus, novel therapies enhancing Filaggrin expression or blocking acquired Filaggrin downregulation were a major target of many clinical trials with promising results [27]. Taken together, it can be suggested that inhibition of OVOL1 could suppress Filaggrin function and discovering OVOL1 agonists may be beneficial in psoriasis treatment.

## Conclusion

In conclusion, OVOL1 and filaggrin might be involved in psoriasis-associated inflammation and skin proliferation. OVOL1 could be a protective barrier in the skin epidermis and its expression in psoriatic epidermis could be used to stratify progressive disease. Filaggrin may have a role in progression of psoriasis. OVOL1 inhibition could be considered in suppression of Filaggrin function. OVOL1 agonists may be beneficial in psoriasis treatment.

## Data Availability

No datasets were generated or analysed during the current study.

## Abbreviations

**PASI**: Psoriasis Area and Severity Index
**IMQ**: imiquimod
**AHR**: aryl hydrocarbon receptor

## References

[1] Arnold KA, Treister AD, Lio PA, Alenghat FJ (2019). Association of Atherosclerosis Prevalence with Age, Race, and traditional risk factors in patients with psoriasis. JAMA Dermatol.

[2] Bu J, Ding R, Zhou L, Chen X, Shen E (2022). Epidemiology of Psoriasis and Comorbid diseases: a narrative review. Front Immunol.

[3] Papp KA, Gniadecki R, Beecker J, Dutz J, Gooderham MJ, Hong CH, Kirchhof MG, Lynde CW, Maari C, Poulin Y, Vender RB (2021). Psoriasis Prevalence and Severity by Expert Elicitation. Dermatol Ther (Heidelb).

[4] Zhou X, Chen Y, Cui L, Shi Y, Guo C (2022). Advances in the pathogenesis of psoriasis: from keratinocyte perspective. Cell Death Dis.

[5] Sbidian E, Chaimani A, Garcia-Doval I, Do G, Hua C, Mazaud C, Droitcourt C, Hughes C, Ingram JR, Naldi L, Chosidow O, Le Cleach L (2017). Systemic pharmacological treatments for chronic plaque psoriasis: a network meta-analysis. Cochrane Database Syst Rev.

[6] Dai X, Schonbaum C, Degenstein L, Bai W, Mahowald A, Fuchs E (1998). The ovo gene required for cuticle formation and oogenesis in flies is involved in hair formation and spermatogenesis in mice. Genes Dev.

[7] Nair M, Teng A, Bilanchone V, Agrawal A, Li B, Dai X (2006). Ovol1 regulates the growth arrest of embryonic epidermal progenitor cells and represses c-myc transcription. J Cell Biol.

[8] Sun P, Vu R, Dragan M, Haensel D, Gutierrez G, Nguyen Q (2021). OVOL1 regulates Psoriasis-Like skin inflammation and Epidermal Hyperplasia. J Invest Dermatol.

[9] Dragan M, Sun P, Chen Z, Ma X, Vu R, Shi Y (2022). Epidermis-intrinsic transcription factor Ovol1 Coordinately regulates barrier maintenance and Neutrophil Accumulation in Psoriasis-Like inflammation. J Invest Dermatol.

[10] Zhang Y, Tu C, Wang S, Xiao S (2018). Expression of skin barrier protein filaggrin in skin diseases without atopic dermatitis. J Biosci Med.

[11] Zhao Y, Terron-Kwiatkowski A, Liao H, Lee SP, Allen MH, Hull PR (2007). Filaggrin null alleles are not associated with psoriasis. J Invest Dermatol.

[12] Hemida AS, Mareae AH, Elbasiony ASA, Shehata WA (2020). Plexin-B2 in psoriasis; a clinical and immunohistochemical study. J Immunoass Immunochem.

[13] Zlobec I, Steele R, Terracciano L, Jass JR, Lugli A (2007). Selecting immunohistochemical cut-off scores for novel biomarkers of progression and survival in colorectal cancer. J Clin Pathol.

[14] Ito T, Tsuji G, Ohno F, Uchi H, Nakahara T, Hashimoto-Hachiya A (2016). Activation of the OVOL1-OVOL2 Axis in the Hair Bulb and in Pilomatricoma. Am J Pathol.

[15] Mócsai G, Gáspár K, Nagy G, Irinyi B, Kapitány A, Bíró T (2014). Severe skin inflammation and filaggrin mutation similarly alter the skin barrier in patients with atopic dermatitis. Br J Dermatol.

[16] Liu J, Xu B, Zheng C, Gong Y, Garibaldi J, Soria D (2019). An end-to-end deep learning histochemical scoring system for breast Cancer TMA. IEEE Trans Med Imaging.

[17] Tsuji G, Hashimoto-Hachiya A, Kiyomatsu-Oda M, Takemura M, Ohno F, Ito T (2017). Aryl hydrocarbon receptor activation restores filaggrin expression via OVOL1 in atopic dermatitis. Cell Death Dis.

[18] Hashimoto-Hachiya A, Tsuji G, Murai M, Yan X, Furue M (2018). Upregulation of FLG, LOR, and IVL expression by Rhodiola Crenulata Root Extract via Aryl Hydrocarbon receptor: Differential involvement of OVOL1. Int J Mol Sci.

[19] Gerritsen MJ, Elbers ME, de Jong EM, van de Kerkhof PC (1997). Recruitment of cycling epidermal cells and expression of filaggrin, involucrin and tenascin in the margin of the active psoriatic plaque, in the uninvolved skin of psoriatic patients and in the normal healthy skin. J Dermatol Sci.

[20] Kim BE, Howell MD, Guttman-Yassky E, Gilleaudeau PM, Cardinale IR, Boguniewicz M (2011). TNF-α downregulates filaggrin and loricrin through c-Jun N-terminal kinase: role for TNF-α antagonists to improve skin barrier. J Invest Dermatol.

[21] Jensen P, Skov L, Thyssen J, Maibach H (2014). Filaggrin in Psoriasis. Filaggrin.

[22] Varma SR, Sivaprakasam TO, Mishra A, Prabhu S, Rafiq M, Rangesh P (2017). Imiquimod-induced psoriasis-like inflammation in differentiated human keratinocytes: its evaluation using curcumin. Eur J Pharmacol.

[23] Smith SH, Jayawickreme C, Rickard DJ, Nicodeme E, Bui T, Simmons C (2017). Tapinarof is a natural AhR agonist that resolves skin inflammation in mice and humans. J Invest Dermatol.

[24] Di Meglio P, Duarte JH, Ahlfors H, Owens ND, Li Y, Villanova F (2014). Activation of the aryl hydrocarbon receptor dampens the severity of inflammatory skin conditions. Immunity.

[25] Furue M, Hashimoto-Hachiya A, Tsuji G (2019). Aryl Hydrocarbon Receptor in atopic dermatitis and psoriasis. Int J Mol Sci.

[26] Furue K, Ito T, Tsuji G, Ulzii D, Vu YH, Kido-Nakahara M, Nakahara T, Furue M (2019). The IL-13-OVOL1-FLG axis in atopic dermatitis. Immunology.

[27] Dębińska A (2021). New treatments for atopic dermatitis targeting skin barrier repair via the regulation of FLG expression. J Clin Med.

